# Framing education on headache disorders into the Global Burden of Disease Study 2010. The European Headache Federation stands ready

**DOI:** 10.1186/1129-2377-14-41

**Published:** 2013-05-10

**Authors:** Paolo Martelletti, Dimos-Dimitrios Mitsikostas, Christian Lampl, Zaza Katsarava, Vera Osipova, Koen Paemeleire, Lars Edvinsson, Aksel Siva, Dominique Valade, Timothy J Steiner, Rigmor H Jensen

**Affiliations:** 1 Department of Clinical and Molecular Medicine, Sapienza University of Rome, Sant’Andrea Hospital, Via di Grottarossa 1035, 00189, Rome, Italy; 2 Department of Neurology, Athens Naval Hospital, Athens, Greece; 3 Department of Neurology and Pain Medicine, Konventhospital Barmherzige Brüder, Linz, Austria; 4 Department of Neurology University of Duisburg-Essen, Essen and Department of Neurology, Evangelisches Krankenhaus Unna, Unna, Germany; 5 Department of Neurology and Clinical Neurophysiology, First Sechenov Moscow State Medical University, Moscow, Russia; 6 Department of Neurology, Ghent University Hospital, Ghent, Belgium; 7 Department of Internal Medicine, University Hospital, Lund, Sweden; 8 Department of Neurology, Cerrahpasa School of Medicine, Istanbul University, Istanbul, Turkey; 9 Hôpital Lariboisière, Paris, France; 10 Department of Neuroscience, Norwegian University of Science and Technology, Trondheim, Norway; 11 Danish Headache Center, Department of Neurology, University of Copenhagen, Glostrup Hospital, Copenhagen, Denmark

## Abstract

Framing education on headache disorders into the Global Burden of Disease Study 2010. The European Headache Federation stands ready.

## Start-up for a new education on headache disorders after Global Burden of Disease Study 2010

The Global Burden of Disease Study 2010 (GBD 2010), recently published in The Lancet [1], has marked new standards for all scientific societies, NGOs and patients’ associations that gravitate towards headache disorders. These standards, dictated not only by the new migraine ranking but also by the appearance of tension-type headache, confirm headache in the top ten causes of global disability, in the top three ranking of global prevalence of disease and at the top of all neurological disorders as causes of years lived with disability. All this has been achieved despite the unjustified absence of medication-overuse headache from GBD 2010. The fundamentals of these results are built on the hard-working epidemiological activity of the Neurologic Disorders Expert Group in Headache for GBD 2010, and *Lifting The Burden* (LTB) [2] which, on the basis of data carefully gathered (and featured in the *Atlas of Headache Disorders and Resources in the World 2011*[3,4]), has then offered to GBD 2010 the headache-specific part of this cultural giant of modern medicine.

What is then the bond between the results therein included in this immense survey, correlated to the terms *disability, sequelae, disability states, years lived with disability, impairments*, and the European Headache Federation (EHF)? It is explicit in the message: “*The principal findings, namely that mental health [impairments] ….. need urgent policy responses, are well established. Monitoring progress in reducing the effect of these, and other major contributors to health loss, is as important for improving population health as monitoring progress against the leading causes of death.”* In the strategy for achieving this in headache, physician education is a *sine qua non*[3,4], upon which quality of care and any multiplicity of structures dedicated to the control of headache depend.

## European Headache Federation educational response

EHF takes pride from its long history of education on primary headache disorders, integral to its mission, “Educate Europe about Headache”. This purpose has been continuously pursued since its foundation over 30 years ago, across both Central and Eastern Europe. Its educational activities have been conducted through the residential EHF Headache Schools, commenced in 2002, and through the more recent distance-learning Video Conference Courses, commenced in 2009. We must also mention the aids offered by EHF in conjunction with LTB and WHO in the management of the most common types of primary headache, the *European principles of management of common headache disorders in primary care*[5], specifically aimed at general practitioners. The efficient penetration of these guidelines has been facilitated by their Italian, Portuguese and Russian translations freely available on the EHF website (http://www.ehf-org.org/).

EHF has also cared about the other, high-academic end of education in headache, through its collaboration with Sapienza University of Rome. The Master in Headache Medicine course, held each year for ten years at Sapienza University of Rome, has been endorsed by EHF since the Academic Year 2005-2006, training a large number of specialists from various European countries and many other geographical areas in the world (http://w3.uniroma1.it/headache) [6,7]. This initiative has allowed the creation of a headache cultural chain following the consolidated *train-the-trainer method* as an achievable foundation for global education [8]. Once trained as headache specialists, physicians will become trainers, offering education in this field to other health-care providers in their own countries. In this way they will give life to the cultural chain, raising awareness wherever they go of headache, its burden and its medical control [9].

EHF has always strongly promoted educational collaborations with other important European Societies, such as European Federation of Neurological Societies (EFNS) and European Federation of Internal Medicine (EFIM). This last collaboration produced a Joint Symposium dedicated to Emergency Headache, with its clinically evident multidisciplinary characteristics and representing 4% of all admissions to the emergency departments of Europe [10]. Recently, the historical partnership between EHF and LTB has been enriched by an *ad hoc* partnership with the European Federation of IASP Chapters (EFIC), creating a Joint Task Force on Headache, which is already active in educating pain physicians and headache experts in some particular headache models.

A further educational product of EHF is the *EHF Headache Series*, pertinent monographs owned by EHF and to be published by Springer, which will cover the emerging sectors of headache science and education.

## Cultural liberalism in headache education allows a better future for headache patients

It is therefore clear that GBD 2010 has found EHF operatively efficient to conduct its educational role in Europe, ready to further the dissemination of fundamental skills in this area of clinical medicine. This position and readiness stand opposite to protectionist self-preservation fostered by an oligarchic culture, and bring new hope to patients who are not yet adequately treated or even yet diagnosed [11].

These fundamental rules of cultural liberalism and collaboration with like-minded neighbouring societies must lead us to set EHF’s educational priorities. Such cultural openness is neither a sign of *captatio benevolentiae* nor a wish for colonization. Headache disorders are and always will be in a multidisciplinary and transversal area, where only those who are capable of integration and development, as opposed to exclusion and disqualification, will achieve and produce positive effects for the immense civil society who need them – for migraine alone, a billion people affected worldwide.

In fact, to produce new evidence in basic sciences and turn it into new molecules or devices for headache has very partial utility, if it does not reach most patients [12]. The use of triptans by only a small minority of people with migraine is exemplary of such failure, and should be a warning. Without better and larger structures dedicated to headache health-care service delivery [13,14], and a concerted educational effort to train the large number of physicians these services require [13-15], few people will benefit, and not necessarily those most in need; the majority will remain without diagnosis and a proper treatment, carrying an impressive personal burden and contributing to that of society, and with not much hope for change.

We must all shrink from this, and reject the closed, exclusive cultural clubs that promise nothing else, obstinately pervaded by ancient self-referenced protectionism.

## Competing interests

The authors declare that they have no competing interests related to this Editorial.

## Authors’ contributions

PM, TJS, RHJ drafted the manuscript. All authors read and approved the final manuscript.
